# Multi-Algorithm Ensemble Learning Framework for Predicting the Solder Joint Reliability of Wafer-Level Packaging

**DOI:** 10.3390/ma18174074

**Published:** 2025-08-30

**Authors:** Qinghua Su, Kuo-Ning Chiang

**Affiliations:** Department of Power Mechanical Engineering, National Tsing Hua University, Hsinchu City 300044, Taiwan; 0967356474shq@gmail.com

**Keywords:** Wafer-Level Packaging (WLP), finite element analysis (FEA), machine learning, ensemble learning

## Abstract

To enhance design efficiency, this study employs an effective prediction approach that utilizes validated finite element analysis (FEA) to generate simulation data and subsequently applies machine learning (ML) techniques to predict packaging reliability. Validated FEA models are used to replace the costly design-on-experiment approach. However, the training time for some ML algorithms is costly; therefore, reducing the size of the training dataset to lower computational cost is a critical issue for ML. Nevertheless, this approach simultaneously introduces new challenges in maintaining prediction accuracy due to the inherent limitations of small data machine learning. To address these challenges, this work adopts Wafer-Level Packaging (WLP) as a case study. It proposes an ensemble learning framework that integrates multiple machine learning algorithms to enhance predictive robustness. By leveraging the complementary strengths of different algorithms and frameworks, the ensemble approach effectively improves generalization, enabling accurate predictions even with constrained training data.

## 1. Introduction

Long-term reliability remains a central concern in electronic packaging, particularly as device miniaturization and functional integration continue to advance. Among advanced packaging technologies, WLP has gained significant traction due to its compact form factor and superior electrical performance, as demonstrated by the study of Chen et al. [1], who highlighted the challenges and prospects for advanced packaging. However, the inherent mismatch in the coefficients of thermal expansion (CTE) between dissimilar materials frequently induces thermomechanical stress, leading to failure in solder joints. Ismail et al. [2] further reviewed the effects of extreme conditions on solder joint reliability and confirmed that CTE mismatch is a primary driver of failure mechanisms under thermal loading. Notably, the solder ball located at the farthest Distance to Neutral Point (DNP) is often the most susceptible to damage.

To evaluate the thermal reliability of electronic packaging, the accelerated thermal cycling test (ATCT) is widely employed by the electronic packaging industry, as highlighted in the work of Bender et al. [3], who discussed modern trends in microelectronics packaging reliability testing. Although experimental validation methods are robust and widely accepted, they are typically associated with high costs and extended time requirements, making them inefficient for iterative design optimization. As a result, FEA has emerged as a valuable tool during the early design stages, offering a computational means to assess reliability performance. For instance, Liu et al. [4] developed a three-dimensional FEA model of a Wafer-Level Chip Scale Package (WLCSP) to estimate the incremental equivalent plastic strain, showing good agreement with experimental data. Likewise, Wu et al. [5] proposed a two-dimensional symmetric half-diagonal model to reduce simulation time while maintaining prediction accuracy.

Despite its advantages, FEA-based simulation requires considerable domain expertise and is prone to variability due to modeling assumptions, mesh sensitivity, and the researcher’s experience. To address these limitations, a data-driven strategy [6] is proposed: generating datasets from validated FEA simulations to train machine learning models for rapid and consistent reliability predictions. This approach minimizes reliance on manual FEA modeling and enables real-time estimation once the AI model is trained. A detailed discussion of how to validate the simulation method will be provided in a later section.

However, generating high-quality datasets still incurs computational costs. Therefore, it is essential to develop machine learning strategies that can effectively operate under the constraint of small data. In this study, ensemble learning, which integrates multiple predictive algorithms and frameworks, offers a promising solution to improve generalization. Su et al. [6] demonstrated the feasibility of using ensemble neural networks trained on small datasets to predict WLP reliability with high accuracy. Building upon this foundation, the present study further investigates ensemble learning architectures that combine diverse machine learning algorithms to enhance prediction performance. In addition, a novel ensemble framework is proposed in this study, where the 1000-cycle threshold serves as a decision boundary to guide the weighting of base models and improve prediction accuracy.

For algorithm selection, beyond the widely adopted Artificial Neural Network (ANN), prior work by Sunil et al. [7] identified additional models with strong reliability prediction capabilities, such as Gaussian Process Regression (GPR), which provides probabilistic predictions with uncertainty quantification, and Recurrent Neural Network (RNN), which captures temporal dependencies in sequential data. Further discussion on the algorithmic mechanisms and the reliability empirical model will be presented in the subsequent section.

**Figure 1:**
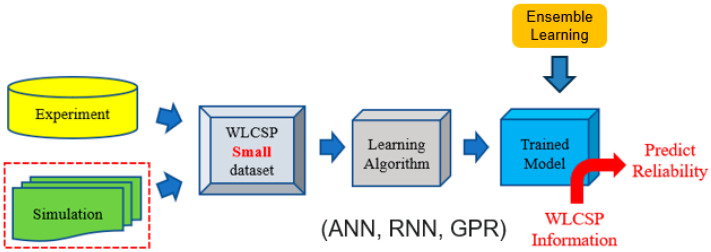
The workflow of AI-assisted design of simulation with a small dataset.

As an overview, Figure 1 outlines the complete workflow developed for small datasets, which serves as the foundation for the discussions in the following sections.

## 2. Fundamental Theory

### 2.1. Coffin–Manson Model

In reliability life prediction of packaging structures, the methodologies are generally categorized into two types: strain-based and energy-based approaches. The present study adopts a strain-based approach, specifically utilizing the Coffin–Manson model. Following FEA, the increment of equivalent plastic strain is extracted, which serves as a critical parameter for estimating the fatigue life of solder joints. The Coffin–Manson relationship is formulated as shown in Equation (1) [8].

(1) $$N_{f}=\alpha {\left({\Delta \varepsilon }_{eq}^{pl}\right)}^{\phi },$$

where $N_{f}$ represents the predicted fatigue life (in cycles), and ${\Delta \varepsilon }_{eq}^{pl}$ denotes the increment of equivalent plastic strain obtained by simulation. In this study, the coefficients α and ϕ, determined through regression analysis, are empirical constants with values of 0.235 and −1.75 [6], respectively.

### 2.2. Artificial Neural Network (ANN)

**Figure 2:**
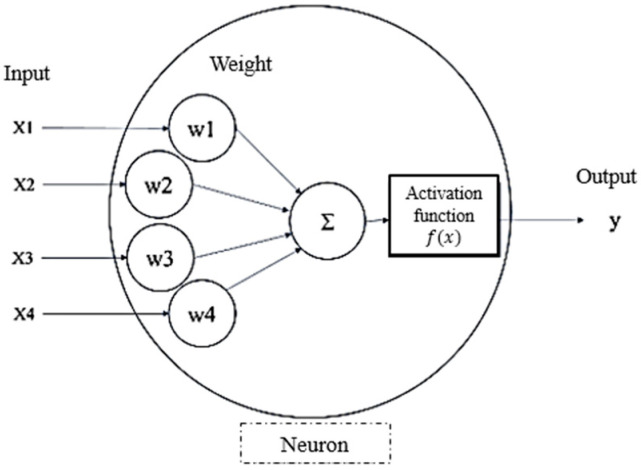
Schematic of a single neuron.

As a classical machine learning model for handling nonlinear regression problems, the ANN is a computational model inspired by the architecture and functioning of biological neural systems. Over the decades, ANN has evolved into modern forms such as multi-layer perceptrons (MLP), which are widely employed as supervised learning models to approximate a target function $f\left(\cdot \right)$. As illustrated in Figure 2, each neuron, which serves as the fundamental unit of the network, receives multiple numerical inputs, each associated with an individual weight. These inputs undergo a weighted summation, and the result is processed through an activation function, which introduces nonlinearity into the model and enables it to capture complex relationships between inputs and outputs. In this study, the Rectified Linear Unit (ReLU) function [9], as defined in Equation (2), is adopted as the fixed activation function, considering both training efficiency and generalization performance, which is particularly beneficial in shallow networks with limited training data.

(2) $$f\left(x\right)=\max\left(0,x\right),\;f^{\prime }\left(x\right)=\left\{\begin{array}{c} 1,\;if\;x>0\\ 0,\;if\;x\leq 0 \end{array}\right.,$$

**Figure 3:**
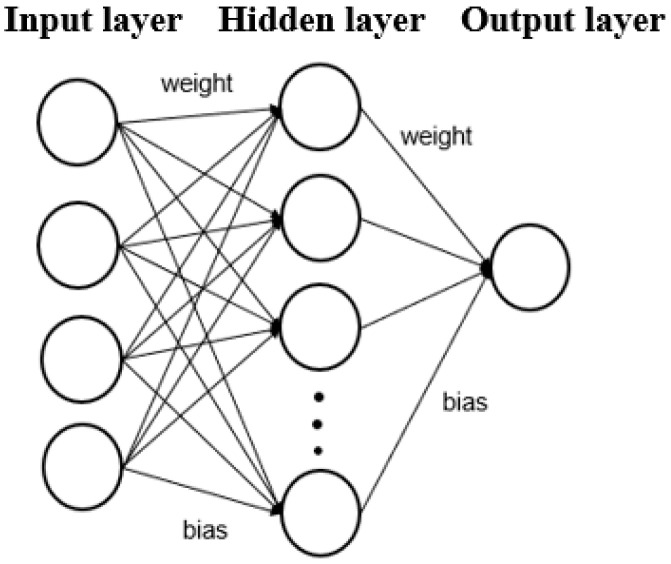
Structure of one hidden layer ANN.

The main components of an ANN include the input layer, hidden layer(s), and output layer. Each layer is composed of neurons, and connections are established between neurons in adjacent layers. Figure 3 illustrates the structure of an ANN with a single hidden layer. This type of structure, where information flows in a single direction (as indicated by the arrows) without any loops or feedback paths, is referred to as a feedforward neural network. The inclusion of bias terms enhances the model’s generalization ability, helping to prevent both overfitting and underfitting.

To train such a network effectively, a learning algorithm is required to adjust the connection weights based on the observed error. One of the most widely adopted algorithms for this purpose is the Backpropagation (BP) algorithm [10], which enables supervised learning by iteratively minimizing the difference between predicted and actual outputs. This process is typically carried out using an optimization method, commonly referred to as a solver, which updates the weights by following the gradient of a defined loss function. For regression problems, the loss is typically measured using the mean square error (MSE), as shown in Equation (3).

(3) $$MSE=\frac{1}{n}\sum_{i=1}^{n}{\left(y_{i}-{\hat{y}}_{i}\right)}^{2},$$

where n is the number of training data, $y_{i}$ presents the target value, and ${\hat{y}}_{i}$ presents the predicted value.

The choice of solver, such as Adaptive Moment Estimation (Adam) or Limited-memory Broyden–Fletcher–Goldfarb–Shanno (L-BFGS), influences the convergence behavior and the final performance of the network.

Adam is a first-order optimization algorithm that adaptively adjusts learning rates using estimates of the first and second moments of gradients. Its computational efficiency and robustness make it well-suited for training deep networks and handling large-scale datasets.

In contrast, L-BFGS is a quasi-Newton method that approximates second-order curvature information to accelerate convergence. It is particularly effective in small-data scenarios and shallow network architectures.

The task addressed in this study aligns well with the characteristics of small-data and shallow-network scenarios. Consistent with this, experimental results indicate that the L-BFGS solver outperforms Adam in terms of training performance [11]. However, to promote model diversity within the ensemble framework, both solvers are employed in this work.

### 2.3. Recurrent Neural Network (RNN)

RNN constitutes a distinct class of neural architecture developed to process sequential data by capturing temporal dependencies and dynamic patterns. RNN incorporates a hidden state that is recursively updated at each time step, allowing the network to preserve and integrate information across different positions in the input sequence. This internal memory structure enables the capture of both long-term and short-term dependencies in data where temporal order or sequential structure is relevant. Although the target task in this study is a conventional, non-sequential regression problem, RNN has still demonstrated strong predictive capability. More importantly, their unique architecture allows them to learn feature representations that differ from those of feedforward models, thereby contributing complementary insights within the ensemble framework and enhancing overall prediction robustness.

**Figure 4:**
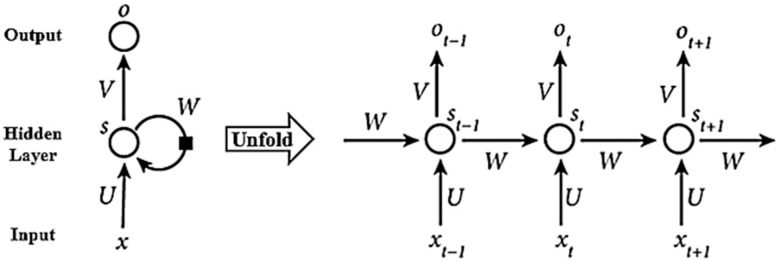
Schematic of RNN structure.

Figure 4 presents a simplified illustration of the RNN structure. The left side of the figure highlights the recurrent connection using a self-loop, while the right side depicts the network unrolled over three consecutive time steps: t−1, t, and t+1.

Equation (4) defines the general expression of the output $o_{t}$ without bias:

(4) $$o_{t}=g\left(V\cdot f\left(U\cdot \;x_{t}+W\cdot \;s_{t-1}\right)\right),$$

where both $g\left(\cdot \right)$ and $f\left(\cdot \right)$ are used as activation functions. U, W, and V denote the weight matrices that remain shared across all time steps in this RNN structure.

**Figure 5:**
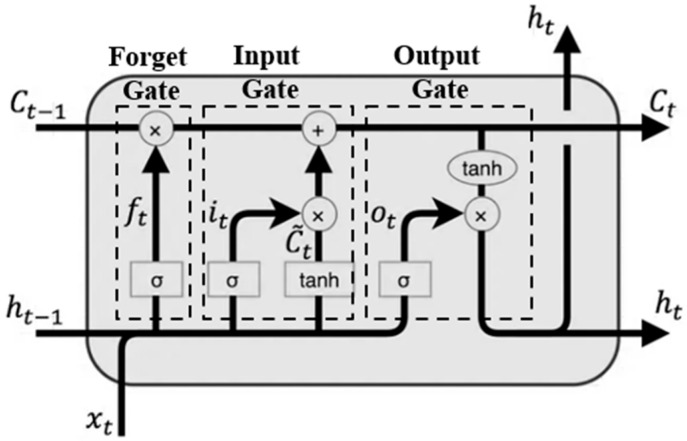
Schematic of an LSTM unit.

This structure is also referred to as the vanilla (simple) RNN unit. It computes the current state value solely based on the input at the current time step and the state value from the previous time step, without incorporating any additional processing mechanisms. While the simple RNN unit provides a foundational framework for processing sequential data by maintaining a hidden state across time steps, it often encounters difficulties in capturing long-term dependencies due to the vanishing and exploding gradient problems during training. To address these limitations, Long Short-Term Memory (LSTM) networks [12] were introduced as an improved recurrent architecture, as shown in Figure 5. LSTM extends the memory capability of RNNs by introducing a memory cell along with three gating mechanisms: the input gate, the forget gate, and the output gate. These components work together to regulate the flow of information over time. This structure enables LSTM networks to selectively preserve or discard information, which makes them particularly effective in capturing long-term dependencies in sequential data.

The update rule is defined in Equations (5)–(10).

(5) $$f_{t}=\sigma \left(W_{f}x_{t}+U_{f}h_{t-1}+b_{f}\right),$$

(6) $$i_{t}=\sigma \left(W_{i}x_{t}+U_{i}h_{t-1}+b_{i}\right),$$

(7) $$\tilde{c_{t}}=\tanh\left(W_{c}x_{t}+U_{c}h_{t-1}+b_{c}\right),$$

(8) $$o_{t}=\sigma \left(W_{o}x_{t}+U_{o}h_{t-1}+b_{o}\right),$$

(9) $$c_{t}=f_{t}⊙c_{t-1}+i_{t}⊙\tilde{c_{t}},$$

(10) $$h_{t}=o_{t}⊙tanh\left(c_{t}\right),$$

where $x_{t}$, $h_{t}$, and $c_{t}$ represent the input, hidden state, and cell state at time step t, respectively. The symbols $f_{t}$, $i_{t}$, and $o_{t}$ correspond to the forget gate, input gate, and output gate. The term $\tilde{c_{t}}$ denotes the candidate cell state. $W_{*}$, $U_{*}$, and $b_{*}$ represent the weight matrices and bias vectors associated with each gate. The function σ denotes sigmoid activation, while tanh represents the hyperbolic tangent function. The operator ⊙ indicates Hadamard multiplication.

As a type of neural network architecture, RNN also adopts a training mechanism based on the backpropagation algorithm [13]. The selection of several key hyperparameters remains consistent with that used in the feedforward ANN model based on a trade-off between prediction accuracy and training efficiency, as supported by empirical observations in this study. Specifically, the ReLU function is employed as the activation function, and the Adam optimizer is adopted. It should be noted that the use of ReLU here refers to the activation applied at the output of each RNN unit, which is distinct from the sigmoid and tanh functions internally used within LSTM units for gate operations.

It is worth emphasizing that, according to the study by Sunil et al. [7], the use of mean absolute percentage error (MAPE) as the loss function yields slightly better performance than MSE under shallow network architectures with a limited number of neurons. Therefore, MAPE is adopted in this study as shown in Equation (11), where all $y_{i}$ values are positive.

(11) $$MAPE=\frac{1}{n}\sum_{i=1}^{n}\frac{\left|y_{i}-{\hat{y}}_{i}\right|\times 100}{y_{i}},$$

Additionally, a single-directional RNN is used to reduce training costs, as bidirectional models require higher computation due to their dual-directional processing. Like the ANN, both simple RNN units and LSTM units are employed in this study to enhance the diversity of the base models.

### 2.4. Gaussian Process Regression (GPR)

As a non-neural network algorithm for training base models, GPR has demonstrated strong performance under a small amount of training data, which is theoretically supported by its Bayesian formulation and the use of kernel functions to reflect prior knowledge about the target function. This allows the model to generalize effectively even with limited training data.

**Figure 6:**
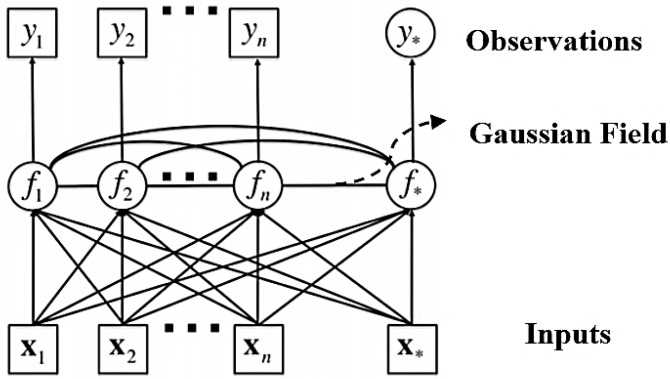
Schematic of GPR.

Unlike parametric models, GPR formulates the regression problem in a fully probabilistic manner by directly modeling the latent function, as illustrated in Figure 6. A major advantage of this approach is its inherent ability to quantify predictive uncertainty through the posterior distribution [14]. GPR assumes that the target function f(x) follows a Gaussian Process prior, while observational noise is modeled as an independent Gaussian distribution, as expressed in Equation (12).

(12) $$y=f\left(x\right)+\varepsilon ,\;f\left(x\right)\;\sim \;GP\left(m\left(x\right),\;k\left(x,x^{\prime }\right)\right)\;and\;\varepsilon \;\sim \;N\left(0,\;\sigma_{n}^{2}\right),$$

where $m\left(x\right)$ is the function, which is typically assumed to be zero, and $k\left(x,x^{\prime }\right)$ is the kernel function that defines the covariance between input points x and $x^{\prime }$.

Let X and y denote the training inputs and their corresponding observations. $X_{*}$ and $y_{*}$ represent the test inputs and predicted outputs derived from the latent function $f_{*}$. The joint distribution of the above parameters is given in Equation (13).

(13) $$\left[\begin{array}{c} y\\ f_{*} \end{array}\right]\;\sim \;N\left[0,\;\begin{array}{cc} K\left(X,X\right)+\sigma_{n}^{2}I & K\left(X,X_{*}\right)\\ K\left(X_{*},X\right) & K\left(X_{*},X_{*}\right) \end{array}\right],$$

where $\sigma_{n}^{2}I$ means a noise term, K denotes the covariance matrix, and 0 denotes the simplified expression of $\left[\begin{array}{c} m(X)\\ m(X_{*}) \end{array}\right]$.

The predictive mean and covariance for the test outputs can be derived from the conditional properties of multivariate Gaussian distributions, as shown in Equations (14) and (15).

(14) $$\bar{f_{*}}=K\left(X_{*},X\right){\left[K\left(X,X\right)+\sigma_{n}^{2}I\right]}^{-1},$$

(15) $$cov\left(f_{*}\right)=K\left(X_{*},X_{*}\right)-K(X_{*},X){\left[K\left(X,X\right)+\sigma_{n}^{2}I\right]}^{-1}K(X,X_{*}),$$

This framework can adapt to the intrinsic structure of the data while simultaneously optimizing its hyperparameters, which are determined by the choice of the kernel function, via maximum likelihood estimation, enabling flexible and precise modeling of complex functions. It is essential to emphasize that the selection of the kernel function has a significant impact on the effectiveness of GPR. In this study, the Matérn kernel and the Radial Basis Function (RBF) kernel are employed, as defined in Equations (16) and (17), respectively.

(16) $$k\left(x_{i},x_{j}\right)=\frac{1}{\Gamma \left(v\right)2^{v-1}}{\left(\frac{\sqrt{2v}}{l}d\left(x_{i},x_{j}\right)\right)}^{v}K_{V}\left(\frac{\sqrt{2v}}{l}d\left(x_{i},x_{j}\right)\right),$$

(17) $$k\left(x_{i},x_{j}\right)=exp(\frac{-{d\left(x_{i},x_{j}\right)}^{2}}{2l^{2}}),$$

where $\Gamma \left(\cdot \right)$ denotes the gamma function, v is the smoothness parameter governing the differentiability of the function, $K_{V}\left(\cdot \right)$ represents the modified Bessel function of the second kind, l is the length scale, and $d\left(\cdot ,\cdot \right)$ refers to the Euclidean distance between input points.

Once the kernel function is defined, the corresponding set of kernel hyperparameters θ to be optimized is also specified in the GPR framework. Although both the GPR and ANN models in this study employ variants of the L-BFGS algorithm for training, their respective optimization objectives differ fundamentally due to the underlying differences in model formulation. For ANN, the training objective is to minimize the MSE between predictions and targets. In contrast, GPR minimizes an objective function as well, where the training process aims to identify the optimal hyperparameters θ by minimizing the negative log marginal likelihood (NLML) [15] of the observed data. The general form of the objective function is given as Equation (18):

(18) $$NLML=-\log\left(y|X,\theta \right)=\frac{1}{2}y^{T}K^{-1}y+\frac{1}{2}\log\det\left(K\right)+\frac{n}{2}log2\pi ,$$

where K denotes the kernel matrix parameterized by θ, and y is the vector of observed targets.

To address this unconstrained optimization task, the “fmin_l_bfgs_b” algorithm from the SciPy library is utilized. This algorithm is a quasi-Newton method that approximates the inverse Hessian and allows for bound constraints, making it well-suited for maintaining kernel parameters within physically meaningful limits.

In practice, a noise term $\sigma_{n}^{2}I$ is also added to the kernel matrix to account for observation noise in the training data, resulting in the modified covariance expression $K+\sigma_{n}^{2}I$. This adjustment not only improves numerical stability but also more accurately captures the uncertainty inherent in real-world measurements. In implementation, this is achieved by specifying the parameter “alpha”, which can be interpreted as the variance of additional Gaussian noise associated with the training targets.

### 2.5. Ensemble Learning

**Figure 7:**
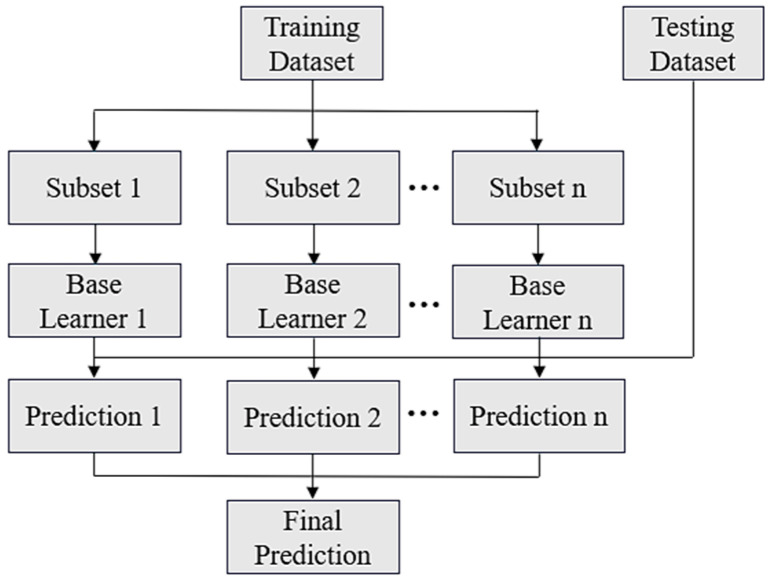
Schematic of Bagging.

**Figure 8:**
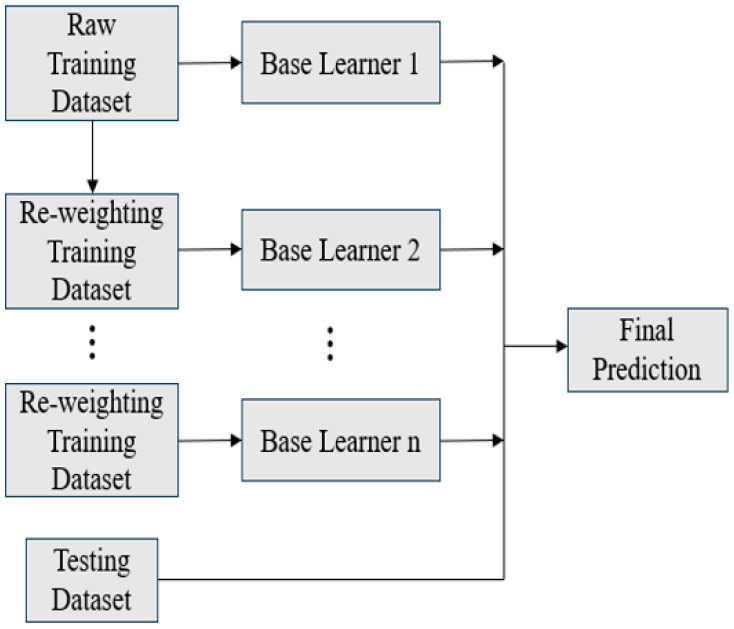
Schematic of boosting.

**Figure 9:**
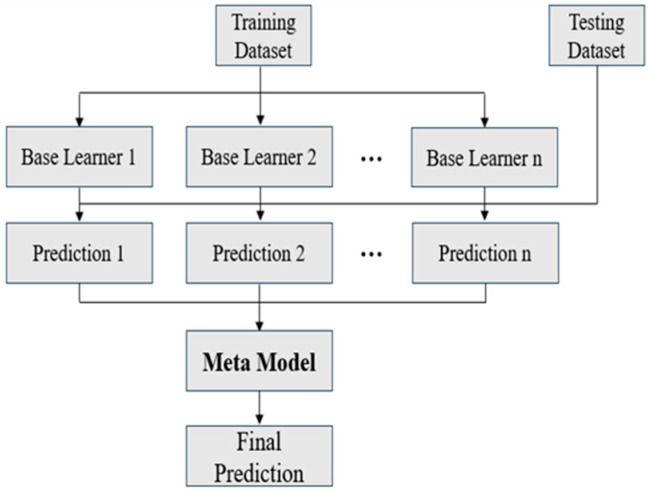
Schematic of stacking.

To mitigate the time cost associated with data generation, training on small datasets is often necessary. However, this constraint can result in unstable predictive performance of individual AI models due to overfitting. Ensemble learning provides an effective approach to improve prediction accuracy and robustness. This study adopts three ensemble strategies, namely Bagging, Boosting, and Stacking [16], as illustrated in Figure 7, Figure 8 and Figure 9.

In brief, Bagging improves prediction by averaging the outputs of multiple base learners, with optional weighting in regression tasks. Boosting assigns higher importance to previously mispredicted data points, which are defined as samples with large prediction errors, during successive training rounds and aggregates predictions through a weighted average. Stacking combines the outputs of several base learners as inputs to a meta-model, which is then trained to generate the final prediction.

In this study, ensemble learning is employed as an essential approach to enhance predictive reliability in small-data scenarios. By combining multiple machine learning models, including ANN, RNN, and GPR, using Bagging, boosting, and stacking strategies, the proposed framework improves accuracy, enhances stability, and reduces the risk of overfitting. These improvements contribute to a more robust and dependable solution for data-driven reliability prediction.

## 3. FEA Validation of WLCSP

To reduce computational cost, a simplified two-dimensional finite element model is employed in this study, with the following fundamental assumptions: all materials are homogeneous and isotropic; the temperature within the structure is uniform; residual stresses are neglected; and perfect bonding is assumed at all material interfaces.

**Figure 10:**
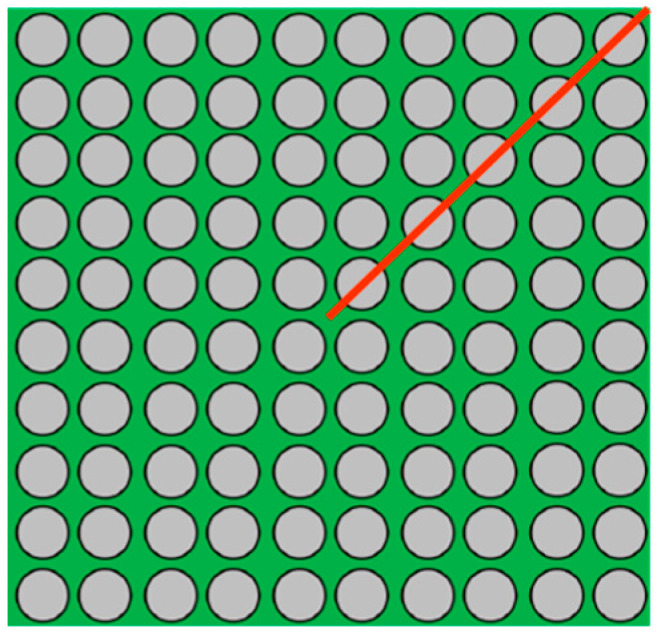
The top view of the WLCSP structure. The red line represents the half-diagonal, and the circles represent solder balls.

**Figure 11:**
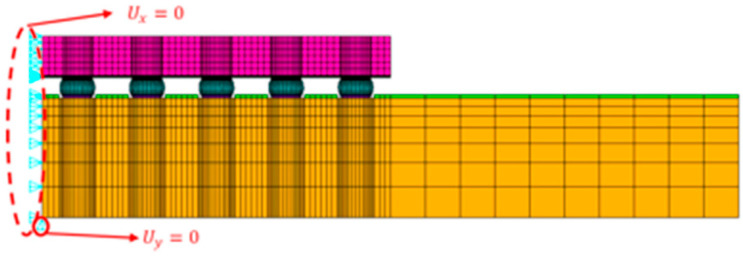
Two-dimensional FEA model for WLCSP.

Let ∆α represent the CTE mismatch between the substrate and the wafer and L denote the DNP of the solder joint. After material parameters are determined, ∆α is treated as a fixed value. The mismatch in thermal deformation between the substrate/PCB and the die at the solder joint is expressed as ΔL=Δα⋅L⋅ΔT. Since this deformation difference increases with distance from the chip center, the outermost corner solder ball typically becomes the most critical location for failure initiation. A top view of the package layout is shown in Figure 10. The red line indicates the half-diagonal direction, and the circles represent solder balls. Figure 11 presents the boundary condition configuration of the FEA model.

**Figure 12:**
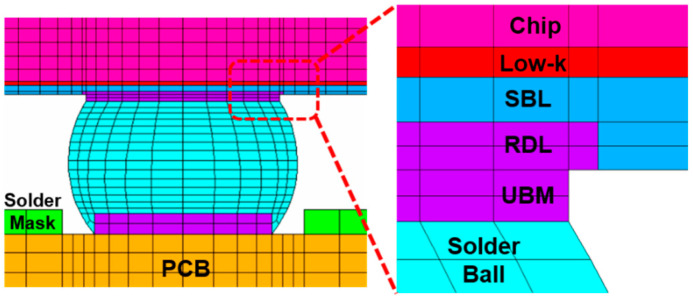
Local view of the FEA model.

The FEA models are constructed according to the geometric specifications of the test vehicles (TVs) [17,18]. The model comprises the silicon chip, low-k layer, stress buffer layer (SBL), under-bump metallurgy (UBM), redistribution layer (RDL), printed circuit board (PCB), copper pad, solder mask, and solder ball, as shown in Figure 12.

**Figure 13:**
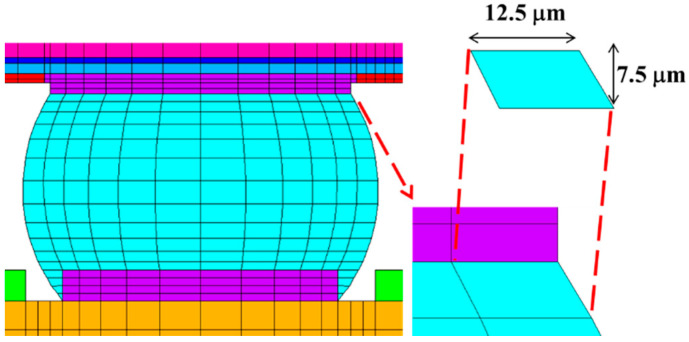
Mesh size in the critical region.

Following the approach proposed by Tsou [2], fixed mesh sizes are applied to critical regions to improve the accuracy and reliability of thermal simulations in the FEA model. As shown in Figure 13, the mesh is refined to 7.5 μm in height and 12.5 μm in width.

**Figure 14:**
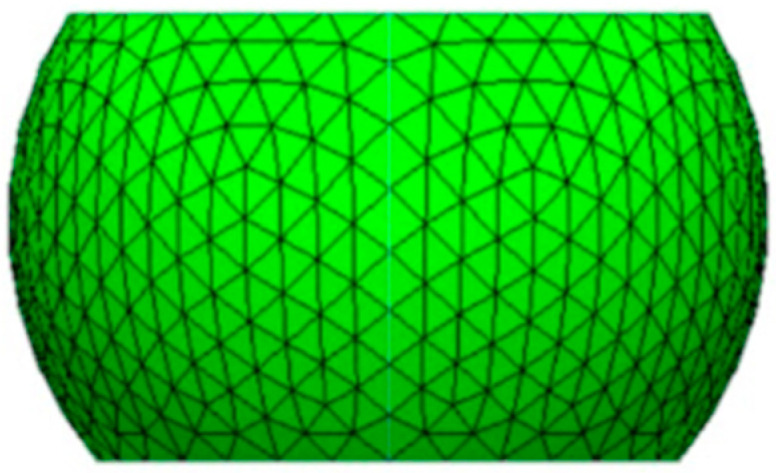
Solder ball shape generated by Surface Evolver.

It should be emphasized that Surface Evolver V2.70 [19] has been employed to estimate the geometric profile of the solder balls after reflow, and the coordinate data of key nodes have been extracted for FEA input. Figure 14 presents the reference geometry.

Apart from specifying the structural geometry and mesh size in the FEA model, material properties represent another key modeling requirement. Except for the solder ball, all materials in the model are assumed to be linear elastic, with their properties listed in Table 1, where T is expressed in degrees Celsius. The solder material used in this study is SAC305, whose Young’s modulus varies with temperature and exhibits pronounced nonlinear mechanical behavior. To characterize this nonlinearity, the Chaboche kinematic hardening model is applied.

**Figure 15:**
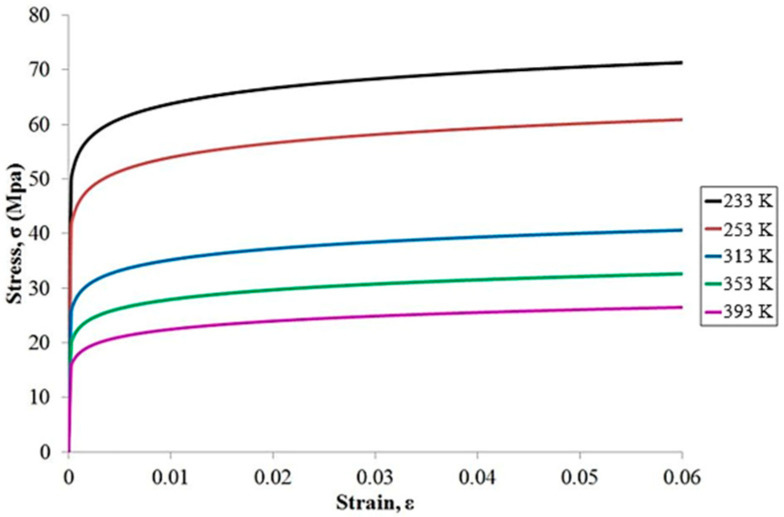
Stress–strain curves for SAC305 at different temperatures.

As shown in Figure 15, uniaxial tensile tests were conducted on SAC305 to obtain its stress–strain responses under different temperature conditions [20]. These experimental results serve as the basis for curve fitting to extract the material parameters required by the Chaboche model.

The corresponding formulation is given in Equation (19) [21]:

(19) $$\alpha =\frac{C}{\gamma }\left(1-e^{-\gamma {\cdot \varepsilon }_{p}}\right)+\sigma_{0},$$

where $\sigma_{0}$ denotes the initial yield stress, with C and γ representing empirical coefficients determined through curve fitting. The corresponding values are listed in Table 2.

**Figure 16:**
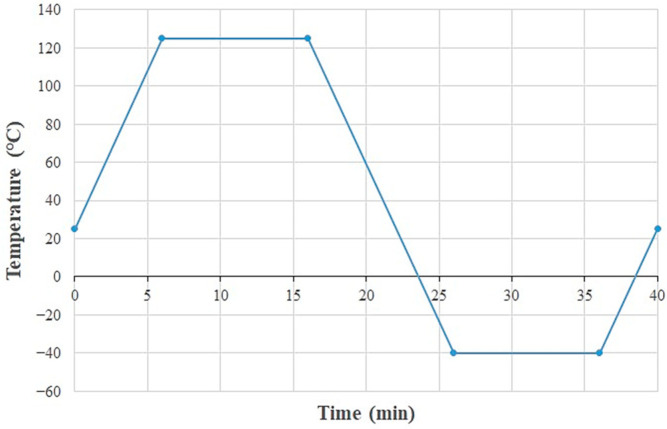
Temperature profile for thermal cycling.

For the loading setup, thermal cycling is applied to the WLCSP model based on JEDEC standard Condition G, with the temperature varying between −40 °C and 125 °C. Each cycle consists of 10 min of dwell time at the temperature extremes and a heating/cooling rate of 16.5 °C per minute, resulting in a total cycle duration of 40 min, as shown in Figure 16.

Upon completing the necessary model setup and preprocessing, the Coffin–Manson equation is then applied to evaluate the simulated reliability of the package using the incremental equivalent plastic strain from the simulation result. Table 3 presents a comparison of the mean time to failure (MTTF) between simulation results and experimental data for five TVs [13,14].

The discrepancies between the two sets of results remain within an acceptable margin, with deviations of less than 10%. This confirms the predictive accuracy of the FEA-based fatigue life estimation.

With the FEA models validated through comparison with experimental results, datasets have been subsequently generated to support machine learning. The database construction is based on fixed material properties, boundary conditions, and thermal loading, while systematically varying the geometric dimensions of the WLCSP structure. This database serves as the foundation for training the AI model.

## 4. Data Sampling and Model Training

### 4.1. Data Sampling

Before data generation, two critical aspects must be addressed to ensure the quality of the training dataset: feature selection and sample distribution. Feature selection relies on domain knowledge or numerical analysis to identify variables that are highly relevant to the prediction target, thereby enhancing both model accuracy and interpretability [22]. Meanwhile, in small-data scenarios, the distribution of samples plays a pivotal role. A well-structured dataset that adequately covers the input space is essential for improving generalization and ensuring reliable predictive performance.

**Figure 17:**
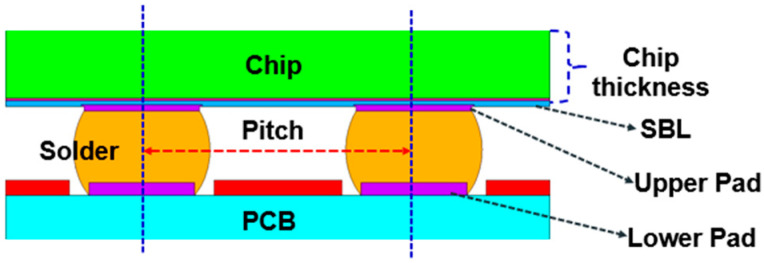
Geometric design factors of the WLP structure.

The key factors influencing solder ball reliability in WLP are identified based on expert knowledge [23,24,25] and are listed in Table 4, along with their corresponding structural locations, as shown in Figure 17.

This study focuses on constructing machine learning datasets based on the four influential parameters: upper pad diameter, lower pad diameter, SBL thickness, and chip thickness. All remaining structural variables are held constant, following the configuration of TV2. Table 5 presents the defined value ranges of the four input features used for generating the dataset.

For data distribution, the training datasets are constructed using a combination of space-filling and adaptive sampling methods [26]. The base models are trained following the standard ensemble neural network procedure outlined by Su et al. [27]. To promote model diversity, each base model is trained using different data volumes and hyperparameter configurations.

Inspired by the concept of boosting, a three-step training process is designed, as summarized in Table 6.

Three datasets, each containing 144 samples, are prepared. In Step 1, Dataset 1 is used for training, and Dataset 2 serves as the test set. In Step 2, mispredicted samples from the previous test set are added to the training set, while Dataset 3 is used for testing. In Step 3, all available data are combined for training without a separate test set. The sampling details of the three datasets were established based on the adaptive sampling procedure proposed by Su et al. [27].

The grid search method [28] is employed at each step, resulting in multiple candidate models. Only the best-performing model is selected from each step. The selection criteria are based on the minimum average test difference in Steps 1 and 2. Due to the absence of testing data, the cross-validation (CV) score is used as the optimal criterion in Step 3. Finally, the generalization capability of the trained models is evaluated using a dataset consisting of 9601 samples [27].

### 4.2. Prediction Results

To ensure consistency among the sub-models, all AI models employ the Robust Scaler, as shown in Equation (20), as the fixed preprocessing method [29]. This preprocessing technique reduces the impact of outliers, improves model robustness, and ensures balanced feature contributions by addressing scale inconsistencies.

(20) $$x^{*}=\frac{x-Q_{2}}{Q_{3}-Q_{1}},$$

where x denotes the raw feature value, $x^{*}$ is the corresponding scaled value, and the quartiles ($Q_{1},\;Q_{2},\;Q_{3}$) are computed from the feature distribution.

**Figure 18:**
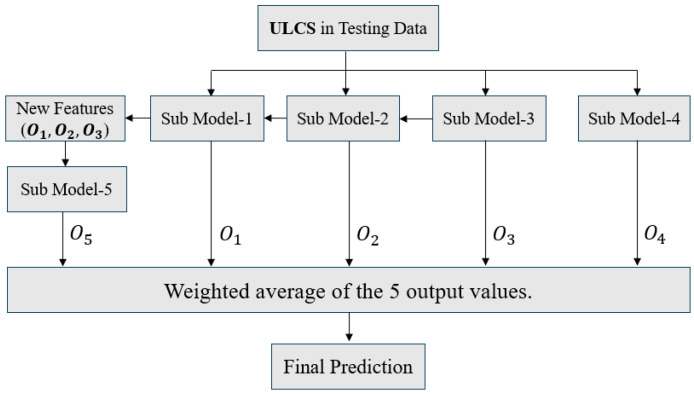
Ensemble structure for a single algorithm.

The three algorithms, namely ANN, RNN, and GPR, are preliminarily combined based on the ensemble structure presented in Figure 18.

The term “ULCS” denotes a set of four geometric structural parameters used as input features in the machine learning model. The optimal models from Steps 1, 2, and 3 are assigned as base models 1, 2, and 3–4, respectively. To enhance model diversity, base model 5 is constructed as a stacking model, which uses the outputs from base models 1 to 3 as its inputs.

Table 7, Table 8 and Table 9 present the key hyperparameter settings used for training the predictive models with the three algorithms. As previously mentioned, to enhance diversity among base models, two solvers (Adam and L-BFGS) are employed for the ANN, two types of recurrent units (LSTM and Simple RNN) are selected for the RNN, and two kernels (Matérn and RBF) are adopted for the GPR.

Additionally, “N_restarts” in Table 9 refers to the number of optimizer restarts, which helps improve model performance by reducing the risk of convergence to suboptimal local minima.

Based on the optimal model selection criteria defined in Table 6, Table 10, Table 11 and Table 12 present the key hyperparameter settings and corresponding model performance for the five base models trained using the three algorithms.

The item “Neuron number” indicates the number of neurons in the three hidden layers. In “Maximum difference”, the listed values represent, in order, the absolute difference, target/prediction, and percentage error.

To improve computational efficiency, the RNN structure assigns the same number of neurons to each hidden layer, thereby significantly reducing the total time required for grid search.

At this stage, all 15 base models used for ensemble learning have been finalized. Their generalization performance is evaluated using a dataset comprising 9601 data points, and the results are summarized in Table 13. The values in parentheses represent, in order, the target value, the predicted value, and the percentage error.

The comparison shows that when the training dataset reaches its maximum size of 432, the average test difference of individual sub-models across all three algorithms remains around 10 cycles.

Overall, the GPR base models underperform compared to those based on ANN and RNN, which also negatively impacts the final ensemble results. The base models from the three algorithms are combined following the structure shown in Figure 18, where the assigned weights are proportional to their respective training set sizes. In this framework, model weights are determined in proportion to the size of their respective training datasets, such that learners trained on larger datasets contribute more strongly to the final prediction. The ensemble models from each algorithm are then equally weighted and further aggregated into a second-level ensemble. The outcomes are presented in Table 14.

The first-level ensembles of ANN and RNN demonstrate notable improvements in prediction performance. In contrast, the ensemble results of GPR are suboptimal, mainly due to the limited accuracy of its individual base models. The second-level ensemble, combining ANN and RNN models, further enhances predictive accuracy, whereas the inclusion of GPR models offers no additional benefit. These results highlight that both model diversity and base-model accuracy are essential to ensemble effectiveness. Ensemble learning becomes truly beneficial only when sufficient predictive accuracy is achieved alongside adequate model diversity. Simply increasing the number of base models does not consistently lead to improved predictive performance.

Thus far, the performance of all ensemble models has been fully demonstrated. The optimal ensemble model achieves an average test error of only seven cycles, along with a notable reduction in the maximum test difference. These results demonstrate the advantage of ensemble learning in improving both the accuracy and stability of predictions. The ensemble not only reduces the average difference but also effectively suppresses extreme deviations, resulting in more reliable and consistent model performance.

The ensemble framework, as illustrated in Figure 18, was designed to facilitate parallel data generation and model training. It adopts a staged training strategy, in which models are iteratively refined based on data generated at each step. Once the training dataset is finalized, the focus naturally shifts from data generation to exploring informative structures within the data that can guide the training of AI models.

**Figure 19:**
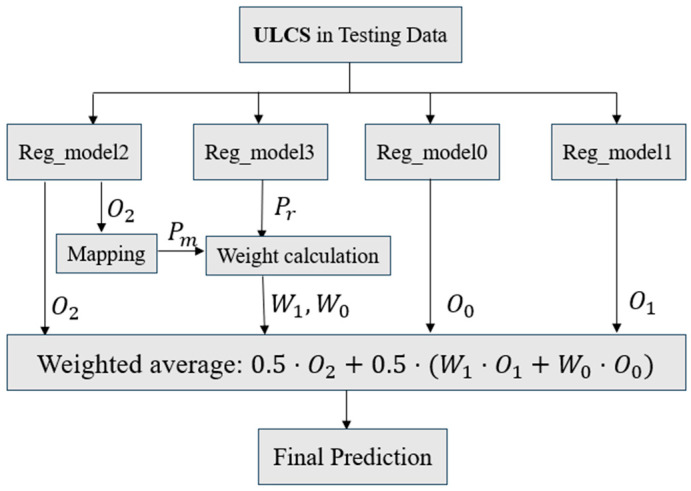
Threshold-based ensemble framework.

In the case of WLP reliability studied here, the 1000-cycle mark [30] is widely recognized as a representative threshold in evaluating WLP reliability. Based on this understanding, a revised ensemble structure can be developed to enhance predictive performance further. The newly proposed ensemble framework is illustrated in Figure 19.

All four AI models in the proposed framework are flexible in algorithm selection. This study only presents results using the ANN. The outputs $O_{0,1,2}$ correspond to three models: Model 0 is trained on samples with lifetimes below 1000 cycles (label 0), Model 1 on samples with lifetimes equal to or above 1000 cycles (label 1), and Model 2 on the entire dataset without threshold-based data partitioning. All AI models are optimized using Grid Search with Cross-Validation to determine the best hyperparameter configurations. The weights, as shown in Figure 19, are defined by Equations (21) and (22).

(21) $$P_{r}=P_{reg_{3}}\left(y=1|x\right),\;P_{m}=clip(\frac{O_{2}-b_{l}}{b_{h}-b_{l}},0,1)$$

(22) $$W_{1}=0.5\cdot \left(P_{r}+P_{m}\right);\;W_{0}=1-W_{1},$$

where $P_{r}$ represents the output $O_{3}$ of “reg_model3”, which maps input features x to a probability value in the range [0, 1], indicating the likelihood that the sample belongs to the high-reliability category (≥1000 cycles). $P_{m}$ is a normalized weight derived from the output $O_{2}$ of the full-data (432) “reg_model2”, scaled linearly between predefined bounds ($b_{l},\;b_{h}$) and clipped to the range [0, 1] to ensure numerical stability. $b_{l}$ and $b_{h}$ denote the lower and upper bounds of the mapping interval as 900 and 1100, respectively. The final ensemble weight $W_{1}$ is computed as the average of $P_{r}$ and $P_{m}$.

The final prediction performance of the two ensemble frameworks is compared in Table 15. In the evaluation using 9601 test samples, the new framework performs slightly better than the previous one. The second-level ensemble of two ensemble frameworks with the same weight further improved prediction performance, reducing the average test difference to 6.5 cycles.

On the other hand, the highest prediction difference consistently occurs at the same data point across two single-algorithm ensemble models, highlighting a key limitation of this approach. Without the introduction of new training data or an improved algorithm, it becomes difficult for models to enhance prediction accuracy on such high-difference samples.

In contrast, the high-error samples tend to differ across algorithms, and integrating multiple algorithms helps offset individual model weaknesses. As a result, multi-algorithm ensembles offer greater potential for improving prediction performance on outlier cases. Nevertheless, the effectiveness of this strategy still relies on the base models achieving sufficient predictive accuracy.

Finally, the ensemble model demonstrating the best overall performance was selected. Using the WLP with design parameters of upper pad diameter (240 µm), lower pad diameter (220 µm), chip thickness (330 µm), and stress buffer layer thickness (7.5 µm) as an example, the differences between the simulation and AI model are compared in Table 16. Once the AI model is trained and finalized, it enables the rapid evaluation of design parameter variations while ensuring consistency and accuracy in prediction results.

## 5. Conclusions

This study examines ensemble learning with multiple algorithms to enhance the reliability prediction of WLP under small-data conditions. The results confirm that ensemble methods can improve predictive performance, particularly when combining models with complementary characteristics. For instance, the ensemble of ANN and RNN achieved good results, demonstrating the value of algorithmic complementarity in addressing data limitations.

However, the findings also reveal that integrating multiple algorithms does not necessarily lead to better outcomes. Although GPR is theoretically well-suited for small-data scenarios, its inclusion consistently reduced ensemble accuracy due to its relatively poor performance on the datasets used in this study. This highlights a key insight: the effectiveness of ensemble learning depends not only on the diversity of models but also on the predictive quality of each base model. Simply increasing the number of sub-models or adding more algorithms can lead to diminishing returns and may introduce issues such as overfitting and increased computational cost.

In addition to algorithm selection, this study highlights the importance of ensemble architecture design. The comparison between two frameworks with distinct structural logic shows that both achieved comparable predictive performance. The second-level ensemble, comprising two ensemble frameworks, further improved prediction performance. These findings suggest that the effectiveness of ensemble learning depends not only on the choice of algorithms but also on how the models are organized and combined.

In summary, ensemble learning remains a promising approach for reducing prediction errors and enhancing generalization, particularly in cases involving small datasets. Future work should focus on improving the accuracy of base models, refining model selection criteria, and developing adaptive ensemble strategies that leverage both algorithmic diversity and architectural flexibility.

## Figures and Tables

**Table 1:** Linear elastic properties used in WLCSP modeling.

Material	Young’s Modulus (GPa)	Poisson’s Ratio	CTE (ppm/°C)
Silicon chip	150	0.28	2.62
Low-k	10	0.16	5
SBL	2	0.33	55
Cu	68.9	0.34	16.7
Solder ball	38.7−0.176T	0.35	25
Solder mask	6.87	0.35	19
PCB	18.2	0.19	16

**Table 2:** Coefficients in Chaboche model.

T(K)	$\sigma_{0}$ (GPa)	C	γ
233	47.64	8894.8	639.2
253	38.87	8573.3	660.0
313	24.06	6011.4	625.2
353	18.12	5804.2	697.7
395	14.31	4804.6	699.9

**Table 3:** Comparison of reliability results for five TVs.

TV	MTTF (Cycle)	Simulation (Cycle)	Difference (Cycle)	Difference (%)
1	318	313	5	1.6%
2	1013	982	31	3.1%
3	587	587	0	0.0%
4	876	804	72	8.2%
5	904	885	19	2.1%

**Table 4:** Ten structural design factors for WLP reliability.

Influence Factors	
Chip Thickness	Chip Size
Upper Pad Thickness	Upper Pad Diameter
Lower Pad Thickness	Lower Pad Diameter
SBL Thickness	PCB Thickness
Solder Diameter	Pitch

**Table 5:** Value range of four features.

**Features**	**Feature Values**
Upper Pad Dia. (Unit: mm)	0.18~0.24
Lower Pad Dia. (Unit: mm)	0.18~0.24
Chip Thickness (Unit: mm)	0.15~0.45
SBL Thickness (Unit: μm)	5~32.5

**Table 6:** Dataset configuration and model selection criteria.

Item	Step 1	Step 2	Step 3
Training data	144	144 + 14	432
Testing data	144	144 + 144	——
Optimal criteria	Test difference	Test difference	CV score

**Table 7:** ANN hyperparameter settings.

Hyperparameter	Setting
Activation function	ReLU
Solver	L-BFGS/Adam
Learning rate	Adaptive
Initial learning rate	0.001
Hidden layers	3
Neuron number	Grid search
Max_iter	5000
Loss function	MSE

**Table 8:** RNN hyperparameter settings.

Hyperparameter	Setting
Activation function	ReLU
Directions	Single
Unit	LSTM/Simple RNN
Learning rate	Adaptive
Initial learning rate	0.001
Hidden layers	3
Neuron number	100/200
Epochs	2000
Loss function	MAPE

**Table 9:** GPR hyperparameter settings.

Hyperparameter	Setting
Kernel function	Matérn/RBF
Alpha	Grid search (1 × 10^−10^~1)
Optimizer	fmin_l_bfgs_b
N_restarts	Grid search (0~20)

**Table 10:** Training results of five ANN-based models.

Item	ANN-1	ANN-2	ANN-3	ANN-4	ANN-5
Training data	144	158	432	432	432
Feature	ULCS	ULCS	ULCS	ULCS	Reliability (3)
Solver	L-BFGS	L-BFGS	Adam	L-BFGS	L-BFGS
Neuron number	88-112-96	104-72-76	112-112-52	88-64-60	56-96-44
Maximum training difference	0 (0)	0 (0)	61 660/721 (9.2%)	6 1327/1333 (0.5%)	31 903/872 (3.4%)
Average training difference	0 (0)	0 (0)	8.3 (0.8%)	0.7 (0.1%)	4.8 (0.5%)
Maximum testing difference	71 1396/1467 (5.1%)	66 1311/1377 (5.0%)	——	——	——
Average testing difference	9.7 (1.0%)	9.0 (0.9%)	——	——	——

**Table 11:** Training results of five RNN-based models.

Item	RNN-1	RNN-2	RNN-3	RNN-4	RNN-5
Training data	144	158	432	432	432
Feature	ULCS	ULCS	ULCS	ULCS	Reliability (3)
Unit	LSTM	LSTM	Simple RNN	LSTM	LSTM
Neuron number	100	200	100	200	200
Maximum training difference	53 1319/1266 (4.0%)	29 1145/1174 (2.5%)	47 1690/1643 (2.8%)	16 1470/1486 (1.1%)	24 1230/1254 (2.0%)
Average training difference	6.1 (0.6%)	5.7 (0.6%)	6.2 (0.6%)	3.9 (0.4%)	5.0 (0.5%)
Maximum testing difference	85 828/743 (10.3%)	64 927/991 (6.9%)	——	——	——
Average testing difference	15.7 (1.7%)	13.3 (1.4%)	——	——	——

**Table 12:** Training results of five GPR-based models.

Item	GPR-1	GPR-2	GPR-3	GPR-4	GPR-5
Training data	144	158	432	432	432
Feature	ULCS	ULCS	ULCS	ULCS	Reliability (3)
Kernel	Matérn	Matérn	RBF	Matérn	Matérn
Alpha	0.01	0.01	0.01	0.01	0.01
N_restarts	11	13	12	19	15
Maximum training difference	23 1690/1667 (1.4%)	20 1319/1299 (1.5%)	50 1690/1640 (3.0%)	21 1225/1204 (1.7%)	53 1321/1268 (4.0%)
Average training difference	4.2 (0.4%)	4.2 (0.4%)	8.7 (0.8%)	3.0 (0.3%)	7.4 (0.7%)
Maximum testing difference	63 609/672 (10.3%)	67 1321/1254 (5.1%)	——	——	——
Average testing difference	14.0 (1.4%)	9.7 (1.0%)	——	——	——

**Table 13:** The performance of the base models on 9601 datasets.

Item	Maximum Testing Difference	Average Testing Difference
ANN-1	102 (1141/1039/8.9%)	13.9 (1.4%)
ANN-2	85 (1595/1680/5.3%)	11.6 (1.1%)
ANN-3	104 (1595/1699/6.5%)	9.0 (0.9%)
ANN-4	86 (689/775/12.5%)	11.9 (1.2%)
ANN-5	78 (1595/1673/4.9%)	9.0 (0.9%)
RNN-1	118 (1237/1355/9.5%)	13.8 (1.4%)
RNN-2	140 (672/812/20.8%)	14.6 (1.5%)
RNN-3	94 (864/770/10.9%)	10.7 (1.0%)
RNN-4	114 (921/807/12.4%)	8.3 (0.8%)
RNN-5	114 (664/778/17.2%)	9.8 (1.0%)
GPR-1	124 (1340/1216/9.3%)	15.2 (1.5%)
GPR-2	86 (1432/1346/6.0%)	12.8 (1.3%)
GPR-3	83 (1340/1257/6.2%)	12.7 (1.2%)
GPR-4	80 (1340/1260/6.0%)	9.8 (0.9%)
GPR-5	82 (1585/1503/5.2%)	11.6 (1.1%)

**Table 14:** The performance of ensemble models on 9601 datasets.

Ensemble	Maximum Testing Difference	Average Testing Difference
ANN	66 (1595/1661/4.1%)	8.1 (0.8%)
RNN	82 (921/839/8.9%)	8.0 (0.8%)
GPR	69 (1097/1028/6.3%)	11.3 (1.0%)
ANN + RNN	**63** (921/858/6.8%)	**7.0 (0.7%)**
ANN + GPR	70 (1097/1027/6.4%)	8.7 (0.9%)
RNN + GPR	77 (1340/1263/5.7%)	8.8 (0.9%)
ALL	67 (1142/1075/5.9%)	7.9 (0.8%)

**Table 15:** Performance comparison of two ensemble frameworks.

Ensemble Framework	Maximum Testing Difference	Average Testing Difference
1 (ANN)	66 (1595/1661/4.1%)	8.1 (0.8%)
2 (ANN)	62 (1595/1657/3.9%)	7.5 (0.8%)
1 + 2 (ANN)	64 (1595/1659/4.0%)	**6.5** (0.6%)

**Table 16:** Performance comparison of the simulation and AI models.

Method	Prediction (Cycles)	Difference from Experiment (Cycles)
Experiment	1013	-
Simulation	982	31 (3.1%)
Machine Learning	1029	16 (1.6%)

## Data Availability

The data presented in this study are available on request from the corresponding author due to project confidentiality concerns.
